# Prospective randomized trial comparing relapse rates in dogs with steroid‐responsive meningitis‐arteritis treated with a 6‐week or 6‐month prednisolone protocol

**DOI:** 10.1111/jvim.17130

**Published:** 2024-06-19

**Authors:** Jeremy H. Rose, Colin J. Driver, Lorna Arrol, Thomas J. A. Cardy, Joana Tabanez, Anna Tauro, Ricardo Fernandes, Imogen Schofield, Sophie Adamantos, Nicolas Granger, Thomas. R. Harcourt‐Brown

**Affiliations:** ^1^ Lumbry Park Veterinary Specialists CVS Group plc Alton United Kingdom; ^2^ Cave Veterinary Specialists Linnaeus group Wellington United Kingdom; ^3^ College of Veterinary Medicine North Carolina State University Raleigh USA; ^4^ Blaise Referrals IVC Evidensia Referrals Birmingham United Kingdom; ^5^ CVS Group plc Diss United Kingdom; ^6^ Paragon Veterinary Referrals Linnaeus group Wakefield United Kingdom; ^7^ Bristol Vet Specialists CVS Group plc Bristol United Kingdom; ^8^ Langford Small Animal Hospital, Bristol Veterinary School University of Bristol Bristol United Kingdom

**Keywords:** aseptic meningitis, immune‐mediated, immunosuppression, neck pain, necrotising vasculitis, PUO, pyrexia of unknown origin

## Abstract

**Background:**

Traditionally, 6‐month courses of prednisolone are used to treat steroid‐responsive meningitis‐arteritis (SRMA), but this medication is associated with adverse effects that can lead to poor quality of life.

**Hypothesis/Objectives:**

Resolution of clinical signs and rate of relapse of SRMA would not be significantly different between a 6‐month prednisolone protocol and a 6‐week protocol.

**Animals:**

Forty‐four hospital cases from multiple referral centers in the United Kingdom (2015‐2019). Twenty of 44 were treated with the 6‐month protocol and 24/44 with the 6‐week protocol.

**Methods:**

Prospective, randomized trial with 12‐month follow‐up. The same prednisolone protocol reinitiated in the event of relapse. Analysis of relapses with binary logistic and Poisson regression modeling.

**Results:**

All cases responded to their treatment protocol. Relapses occurred in 6/20 (30%) of the 6‐month protocol and 9/24 (38%) of the 6‐week protocol. There was no statistical difference in the incidence risk of at least 1 relapse between the 2 groups (odds ratio = 1.40; 95% confidence interval [CI], 0.40‐4.96, *P* = 0.60). Among the 15 dogs that relapsed, 10/15 (67%) relapsed once, 3/15 (20%) relapsed twice, and 2/15 (13%) relapsed 3 times. No statistical difference was detected in the incidence rate ratio (IRR) of total relapse events between the 2 groups (IRR = 1.46; 95% CI, 0.61‐3.48; *P* = 0.40).

**Conclusions and Clinical Importance:**

“Short” 6‐week prednisolone protocols could be used to treat SRMA, thereby presumably reducing the duration and severity of prednisolone's adverse effects.

**Abbreviations**
CI: confidence interval
CSF: cerebrospinal fluid
ESS: English springer spaniels
IQR: interquartile range
OR: odds ratio
QoL: quality of life
SD: standard deviation
SRMA: steroid‐responsive meningitis‐arteritis
TNCC: total nucleated cell count

## 1 INTRODUCTION

Steroid‐responsive meningitis‐arteritis (SRMA) is a well‐recognized and relatively common systemic inflammatory disease of dogs that particularly affects the leptomeninges and its associated arteries.^1^ SRMA can occur in any breed, but beagles, border collies, boxers, English springer spaniels (ESS), Jack Russell terriers, Weimaraners, and whippets show increased chances of developing the disease.^2^, ^3^, ^4^ Bernese mountain dogs, German shorthair pointers, Nova Scotia duck tolling retrievers, petit basset griffon Vendéen, and wirehaired pointing griffons are predisposed, whereas it is controversial in golden retrievers.^3^, ^5^, ^6^, ^7^, ^8^, ^9^, ^10^ The age of onset of SRMA is from 3 months to 9 years but is typically between 6 and 18 months.^5^, ^11^, ^12^ SRMA equally affects male and female dogs, although there might be a predisposition for male dogs.^2^, ^5^, ^12^ Acute and chronic forms of SRMA exist and both present with a waxing and waning disease course. There is no definitive ante‐mortem test for SRMA and diagnosis is based on clinical criteria, laboratory findings, exclusion of other diseases, and response to treatment.^4^ The etiopathogenesis of SRMA is unknown, but an immune‐mediated process is responsible for, at least part, of the pathophysiology.^13^ SRMA is therefore typically treated with immunosuppressive agents, but mild forms of the disease could be treated with nonsteroidal medications.^5^ Prognosis is generally good with the majority of cases responding to immunosuppression. Relapses of the disease are common, occuring in 8%‐60% of diagnosed dogs and can occur during or after immunosuppressive treatment.^4^, ^5^, ^10^, ^14^, ^15^, ^16^

A 6‐month tapering prednisolone regimen is often suggested to treat SRMA.^5^ This duration of prednisolone course is based on concern regarding early cessation of treatment and development of a protracted disease form that might become untreatable.^17^ Glucocorticoids have well‐known adverse effects in SRMA dogs.^10^ The severity of these adverse effects significantly affects quality of life (QoL) and QoL is significantly lower in SRMA dogs while being treated with prednisolone compared with when in clinical resolution.^10^ As a result, combination immunosuppressive protocols have been suggested to allow a shorter duration and reduced dosage of prednisolone.^15^ Prospective studies appraising treatment plans for either efficacy or rate of relapse are lacking for SRMA.

The aim of our study was to compare a “long” 6‐month tapering prednisolone protocol with a “short” 6‐week tapering prednisolone protocol. Our hypothesis was that the “short” prednisolone course would achieve similar resolution of clinical signs per bout of SRMA and have similar rate of relapse of the disease. If our hypothesis is correct the shorter course of prednisolone treatment could be used for SRMA cases in the future, thereby reducing the adverse effects of prednisolone treatment, which affects these dog's QoL.

## 2 MATERIALS AND METHODS

Multicenter, with allocation concealment, prospective, randomized clinical trial with ethical approval from Animal Welfare and Ethical Review Body, University of Bristol VIN/15/012.

### 2.1 Case selection and definitions

Cases were presented to Langford Veterinary Services, United Kingdom; Cave Veterinary Specialists, United Kingdom; Highcroft Veterinary Referrals, United Kingdom; or Fitzpatrick Referrals, United Kingdom, between April 1, 2015 and April 15, 2019. Cases were diagnosed with SRMA and included in the study if dogs had a signalment, history, clinical exam, and clinicopathological features consistent with SRMA and dogs responded to corticosteroids.

In particular, included dogs must have (1) an acute onset or waxing waning history of spinal pain; (2) either a neutrophilic pleocytosis on CSF analysis (without pathological organisms) if the clinical signs were < 7 days duration or a neutrophilic, mixed, or mononuclear pleocytosis on CSF analysis (without pathological organisms) if the clinical signs were >7 days duration; and (3) spinal imaging that excluded other differentials.^4^, ^5^, ^17^ This was either normal orthogonal spinal radiographs or magnetic resonance imaging or computed tomography that were normal or consistent with local inflammation in the meninges or soft tissues surrounding the vertebral column.^12^, ^18^ Cases were excluded if they had (1) neurological deficits other than those previously described for SRMA; or (2) did not have the required 12‐month follow up.^4^, ^5^, ^17^, ^19^

Spinal pain was defined as displaying a behavioral change (eg, vocalization or attempting to bite), or increased resistance to movement, when the spine was manipulated or compressed in various directions. Neutrophilic pleocytosis, in CSF, was defined as a total nucleated cell count (TNCC) >5 cells/mm^3^ with >50% of the TNCC being neutrophils.^20^, ^21^ Mixed pleocytosis was defined as no cell type having a predominance with TNCC >5 cells/mm^3^, and mononuclear pleocytosis as a TNCC >5 cells/mm^3^ with >50% of mononuclear cells.^20^

### 2.2 Procedures

**FIGURE 1:**
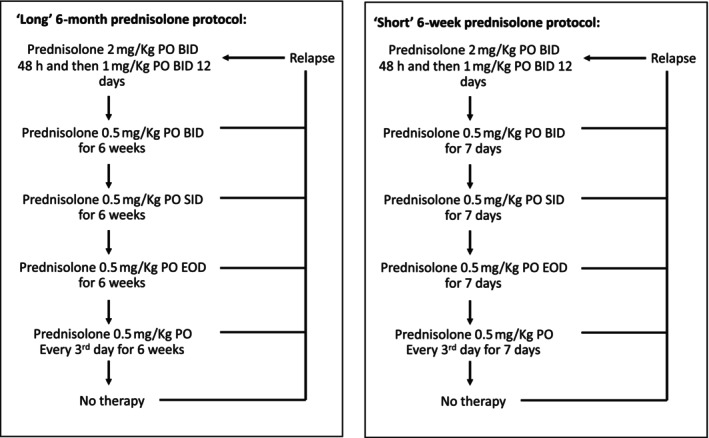
Six‐month and 6‐week treatment schedules.

Once diagnosed with SRMA, cases were randomly assigned to 1 of the treatment strategies by blindly taking a colored counter from a bag containing equal numbers of pink and purple counters. Removal of a pink counter lead to a “long” 6‐month tapering prednisolone dose PO and purple counters lead to the “short” 6 week tapering prednisolone protocol. The colored counters were then replaced back into the bag to maintain a 50 : 50 chance of selection for the next randomization. The prednisolone protocols were given in Figure 1.

Owners were contacted at 10 to 14 days after instigation of treatment to ensure remission of clinical signs. Nonimproved cases were reviewed and taken for further diagnostics or received further immunosuppression if necessary.

Relapse of SRMA was defined as a recurrence of clinical signs that resolved completely after repeated treatment. As a minimum, the relapsing dog had to display neck pain and have their signs diagnosed as compatible with SRMA on clinical assessment by a veterinarian at either their referring practice or referral center. After relapse, the dog was restarted on their initial treatment protocol from the beginning, for example, if they received the 6‐week protocol previously then this 6‐week protocol was given again at relapse, and if they relapsed during tapering of a protocol then they restarted the same protocol from the beginning. The relapse events were recorded for 12 months from diagnosis. Referring vets and owners were asked to contact us to report veterinarian confirmed relapses and compliance of recording relapses was checked by reviewing history from the referring vets for 12 months from diagnosis.

We estimated 40 dogs would need to be enrolled in our study, based on a power calculation assuming *P* = 0.8, α = 0.05, and δ = 0.4 for a relapse rate of 30% using a noninferiority trial and Chi‐squared tests comparing the relapse rate of the differing treatment protocols.

Information regarding age at diagnosis, breed, sex, neuter status, maximum temperature recorded from onset of clinical signs resulting in diagnosis of SRMA, site of CSF collection, CSF result (TNCC, pleocytosis type, and percentage of neutrophils), prednisolone protocol, number of relapses over 12‐month period, number of relapses on treatment, and number of relapses off treatment was recorded for each case.

### 2.3 Statistical analysis

Descriptive demographic and clinical data were presented. Where both cisternal and lumbar CSF samples were taken from a dog the sample with the highest TNCC, and its prospective neutrophil percentage were used. Continuous data were assessed for normality graphically and using Shapiro‐Wilk tests. Median, interquartile range (IQR), and range were reported for non‐normally distributed data, and mean and standard deviation (SD) were reported for normally distributed data. Categorical data were presented showing the count and percentage.

The randomization of characteristics for the 2 treatment groups were compared using *t*‐tests for continuous variables if normally distributed and Wilcoxon rank‐sum tests for non‐normally distributed variables. Comparison of categorical variables were made using Chi‐squared tests and Fisher's exact tests for variables with less than 5 observations in a category. The association between the 2 treatment groups and the incidence risk of at least 1 relapse was assessed using binary logistic regression modeling. The association between the 2 treatment groups and the total count of relapse events per case (incidence rate) was assessed using Poisson regression modeling. Possible confounding factors considered from the current literature were explored within the regression models to examine for substantial changes in the parameter estimates (>10%).

Incidence risk was the proportion of dogs with at least 1 occurrence of relapse recorded within the 12 month follow up. Incidence rate was calculated as the total relapse events recorded across the dogs in the study from the 12‐month period. Incidence rate was reported as events/100 dog‐years.

Statistical significance was set at *P* < 0.05 throughout. Statistical analyses were performed using Stata 17.0 (StataCorp, TX, 2021).

## 3 RESULTS

In total, 45 dogs were enrolled in our study, with 1 being excluded because of lack of follow up, leaving 44 cases for statistical analysis. Median age of dogs was 10 months (IQR, 8‐13; range, 3‐43). The most represented breeds were the beagle (n = 6; 14%), whippet (5; 11%), boxer (4; 9%), and ESS (4; 9%). The remaining breeds were crossbreeds (6; 14%) and other purebreds (19; 43%). There were 31 (70%) male dogs, of which 12 (39%) were neutered, and 13 (30%) female dogs, of which 6 (46%) were neutered. Median maximum recorded temperature, from onset of signs, was 40.1°C (IQR, 39.5‐40.3; range, 38.3‐40.7) with only 1 dog not showing pyrexia (where pyrexia was defined as a temperature >39.2°C).

CSF taps were performed at a cisternal site (n = 29; 66%), lumbar site (8; 18%), or both cisternal and lumbar sites (7; 16%). CSF results indicated neutrophilic pleocytosis in 38 (86%) and a mixed pleocytosis in 6 (14%). In cases where both cisternal and lumbar CSF were taken, the same pleocytosis was obtained from both sampling sites in all cases. Median CSF TNCC was 520 (IQR, 79‐6520; range, 7‐2.00 × 10^12^) and neutrophil percentage was 70% (IQR, 64‐85; range, 35‐96). Neutrophil percentage was not available for 1 dog in the “short” 6‐week tapering protocol as this number was not recorded in the pathologist's report. This was excluded from analysis.

Of the 44 dogs included, 20 (45%) were randomly allocated to receive a “long” 6‐month tapering protocol and 24 (55%) received a “short” 6‐week tapering protocol. There were no significant differences between the case characteristics within each treatment group enrolled in the study (Table 1).

**TABLE 1:** Characteristics of dogs allocated to both treatment protocols.

Variable	Category	6‐month protocol (n = 20)	6‐week protocol (n = 24)	*P*‐value
Breed	Beagle	3 (15%)	3 (13%)	0.40
	Whippet	1 (5%)	4 (17%)	…
	Boxer	3 (15%)	1 (4%)	…
	Springer spaniel	3 (15%)	1 (4%)	…
	Other	10 (50%)	15 (63%)	…
Age (months)	Median (IQR)	12 (9‐16)	10 (7‐12)	0.11
Sex and neuter status	FE	2 (10%)	5 (21%)	0.62
	FN	2 (10%)	4 (17%)	…
	ME	9 (45%)	10 (42%)	…
	MN	7 (35%)	5 (21%)	…
CSF site	Lumbar	3 (15%)	5 (21%)	0.91
	Cisternal	14 (70%)	15 (63%)	…
	Both	3 (15%)	4 (17%)	…
CSF pleocytosis	Neutrophilic	18 (95%)	19 (80%)	0.21
	Mixed	1 (5%)	5 (21%)	…
CSF TNCC	Median (IQR)	1772 (130‐630 000 000)	411 (45‐1421)	0.15
CSF neutrophil percentage	Median (IQR)	77 (66‐85)	70 (60‐82)	0.44
Temperature (max°C)	Median (IQR)	39.8 (39.5‐40.2)	40.1 (39.4‐40.3)	0.42

*Note*: Characteristics of dogs randomly allocated to either the 6‐month or 6‐week tapering prednisolone protocol for the management of steroid‐responsive meningitis‐arteritis (SRMA) (n = 44).

Abbreviations: °C, degree Celsius; CSF, cerebrospinal fluid; FE, female entire; FN, female neutered; IQR, interquartile range; ME, male entire; MN, male neutered; TNCC, total nucleated cell count.

Overall, 15 dogs had at least 1 relapse reported resulting in an incidence risk of 34% (95% confidence interval [CI], 21%‐50%). Relapses occurred in 6/20 (30%) dogs allocated to a 6‐month tapering protocol and 9/24 (38%) allocated to a 6‐week tapering protocol (Table 2). There was no statistical difference in the incidence risk of at least 1 relapse between the 2 tapering protocol groups (odds ratio [OR] = 1.40; 95% CI, 0.40‐4.96, *P* = 0.60).

**TABLE 2:** Incidence of at least 1 relapse for both treatment protocols.

Group	Number with at least 1 relapse	95% confidence interval of %	*P*‐value
6‐month protocol (n = 20)	6 (30%)	12‐54	0.60
6‐week protocol (n = 24)	9 (38%)	19‐59	…

*Note*: Summary of incidence of at least 1 relapse for the 2 tapering prednisolone protocols in dogs with steroid‐responsive meningitis‐arteritis (n = 44).

Among the 15 dogs with relapses, there were 22 events of relapse with 10/15 (67%) relapsing once, 3/15 (20%) dogs relapsing twice and 2 (13%) dogs relapsing 3 times within 12 months. Overall, the incidence rate of relapse events was 50 events/100 dog‐years (Table 3), that is, for every 100 dogs observed in a year, 50 relapses would be expected to occur. In the 6‐month treatment group, 8 events were recorded resulting in an incidence rate of relapse events of 40 events/100 dog‐years. In the 6‐week treatment group, 14 events were recorded resulting in an incidence rate of relapse events of 58 events/100 dog‐years. There was no statistical difference in the incidence rate of relapse events between the 2 tapering protocol groups (incidence rate ratio [IRR] = 1.46; 95% 0.61‐3.48; *P* = 0.40).

**TABLE 3:** Incidence rate of relapse for both treatment protocols.

Group	6‐month protocol (n = 20)	6‐week protocol (n = 24)	Overall (n = 44)
Events of relapse	8	14	22
Dog‐years	20	24	44
Incidence rate of relapse events	40 (events/100 dog‐years)	58 (events/100 dog‐years)	50 (events/100 dog‐years)

*Note*: Summary of incidence rate of relapse for the 2 tapering prednisolone protocols in dogs with steroid‐responsive meningitis‐arteritis (n = 44).

Of the 22 relapse events, 8 (36%) occurred in 6 dogs on treatment and 14 event (64%) occurred in 10 dogs not on treatment. There was no difference between protocol groups and the incidence of at least 1 recorded relapse while on treatment (*P* = 0.39) or off treatment (*P* = 0.30).

The CSF TNCC (*P* = 0.66) and CSF neutrophil percentage (*P* = 0.76) were nonsignificantly associated with the occurrence of at least 1 relapse event. The type of pleocytosis identified on CSF results was also nonsignificantly associated with the occurrence of at least 1 relapse event (*P* = 0.06), with 4/6 (66.7%) of dogs with a mixed pleocytosis relapsing compared with 10/26 (27.8%) of those with a neutrophilic pleocytosis.

## 4 DISCUSSION

In this prospective study, SRMA dogs treated with either the “short” 6‐week or “long” 6‐month tapering prednisolone protocols all clinically responded to their treatment by day 10‐14. There was no significant difference between the “short” 6‐week or “long” 6‐month treatment protocols in the incidence risk of at least 1 relapse event or incidence rate of total relapse events over a 12‐month follow up.

There are no tests to definitively identify relapses of SRMA. Relapses can be diagnosed using clinical criteria and response to treatment alone, or these can be supported with additional diagnostic tests.^14^ Additional diagnostics, such as increased serum C‐reactive protein and IgA CSF levels, are often correlated to relapse of SRMA, but a CSF pleocytosis is rare.^4^, ^10^, ^22^ In our study, at the request of owners, most relapses were clinically confirmed by veterinarians at the referring practices. This was our previous clinical experience and so additional diagnostics were not a requirement of identifying relapse in this “pragmatic trial.”^23^ This was in an effort to limit the exclusion of cases from follow up, and to minimize false negative results, but this could have led to false positive relapses, which could have influenced our results.

In our study, dogs were randomized with allocation concealment to the treatment protocols, but the clinicians were then not blinded. Although randomization should evenly distribute false‐positive relapses between the 2 treatment protocols, the lack of blinding can result in some observer bias. Owners were not made aware of the 2 treatment options and had to seek veterinary assessment to confirm a relapse; in addition, the majority of relapses were confirmed by veterinarians at the referring practice who did not have a known vested interest in the study. As such observer bias could have been low, but this could have affected our results.

Lack of association between relapse of SRMA and prednisolone duration or dose is controversial.^10^, ^12^, ^17^ Six‐month tapering prednisolone protocols are suggested to treat SRMA because of concern that early cessation of treatment is associated with a protracted disease form that becomes untreatable.^12^, ^17^ Protracted disease form can be associated with meningeal fibrosis, which causes ischemic insults to the central nervous system parenchyma, or rarely alters CSF flow and results in hydrocephalus.^1^, ^17^ The “short” 6‐week prednisolone protocol, in our study, was not clinically associated with development of protracted disease form that was untreatable in any case. Review for relapse was only over a 12‐month period in our study, and this might limit ability to discover the protracted disease form or demonstrate a difference in relapse rates between the 2 treatment protocols, as relapses can be prevalent 24 months after diagnosis.^10^

Relapse rates of SRMA in our study, of 34% within a 12 months period from diagnosis, are comparable to reported relapse rates (8%‐60% of cases).^4^, ^5^, ^10^, ^14^, ^15^, ^16^ Relapse rates might be lower in our study, compared with others, given the time frame we monitored for relapse or potential lack of reporting of relapses from referring veterinarians. Relapse rates in our study might be higher, compared with some, given its prospective nature, relapses being identified without the necessity for further diagnostics such as C‐reactive protein to support a SRMA diagnosis, and the treatment protocols selected. It is suggested, in 2 retrospective studies, that their low relapse rates could be associated with combination protocols for treating SRMA, but this is controversial.^3^, ^10^, ^15^, ^16^ It could be that combination protocols will reduce the relapse rate beyond prednisolone alone but this needs further follow up with prospective studies.

The frequency of SRMA relapse and CSF findings are controversial with instances of nucleated cell count being found to be associated with relapse in some studies.^3^, ^10^ CSF TNCC and neutrophil percentage was not statistically associated with relapse in our study. Similarly, no significant difference in relapse rates among cases presenting with either a neutrophilic or mixed pleocytosis on CSF was found. Given the small numbers of dogs with a mixed pleocytosis on CSF analysis in our study, further prospective assessment of the CSF results might be warranted. This might allow identification of cases that require longer or different types of immunosuppressive protocol to avoid a potential refractory form of the disease.

In conclusion, given there was no significant difference between the “short” 6‐week or “long” 6‐month treatment protocols in the incidence risk of at least 1 relapse event or incidence rate of total relapse events in our study, both a “long” 6‐month or “short” 6‐week prednisolone protocol could be considered to treat SRMA cases. “Short” prednisolone protocols would presumably reduce the time and severity of adverse effects from prednisolone in dogs with SRMA. This could limit prednisolone's adverse effects on these dog's QoL. Because QoL assessments were not performed in our study a follow up assessing these values would need to be done to definitively prove this theory.

## CONFLICT OF INTEREST DECLARATION

Authors declare no conflict of interest.

## OFF‐LABEL ANTIMICROBIAL DECLARATION

Authors declare no off‐label use of antimicrobials.

## INSTITUTIONAL ANIMAL CARE AND USE COMMITTEE (IACUC) OR OTHER APPROVAL DECLARATION

Authors declare no IACUC or other approval was needed.

## HUMAN ETHICS APPROVAL DECLARATION

Authors declare human ethics approval was not needed for this study.
